# Familial hypercholesterolemia in Chinese patients with premature ST-segment-elevation myocardial infarction: Prevalence, lipid management and 1-year follow-up

**DOI:** 10.1371/journal.pone.0186815

**Published:** 2017-10-31

**Authors:** Ranshaka Auckle, Binjie Su, Hailing Li, Siling Xu, Mujin Xie, Yangchun Song, Mohammed Abdul Quddus, Yawei Xu, Ban Liu, Wenliang Che

**Affiliations:** 1 Department of Cardiology, Shanghai Tenth People's Hospital, Tongji University School of Medicine, Shanghai, China; 2 Department of Cardiology, Shanghai Tenth People's Hospital Chongming branch, Shanghai, China; Beijing Key Laboratory of Diabetes Prevention and Research, CHINA

## Abstract

**Background:**

Familial hypercholesterolemia (FH), characterized by elevated plasma low-density lipoprotein-cholesterol (LDL-C) levels and premature coronary artery disease (CAD), remains mostly underdiagnosed and undertreated. We investigated the prevalence of clinical FH among Chinese patients with premature ST-segment-elevation myocardial infarction (STEMI) and one-year follow-up on their lipid management and cardiovascular events.

**Methods:**

Four hundred and ninety-eight premature STEMI patients (363men) were enrolled. FH patients were identified using the Dutch Lipid Clinic Network Criteria. Lipid management and cardiovascular events in all patients were assessed.

**Results:**

Nineteen patients (3.8%) were diagnosed as definite/probable FH, 211 (42.4%) as possible FH and 268 (53.8%) as unlikely FH. All patients were divided into two main groups: unlikely FH (0–2 points) and possible FH (≥3 points). Possible FH patients were younger (50.1 years vs. 53.5 years) with higher NT-proBNP level (3014.15 pg/mL vs. 2326.25 pg/mL), occurrence of multi-vessel CAD (37.4% vs. 18.3%), lower LVEF (47% vs. 49%) and more severe Killip classification (Class 3, 20.0% vs. 9.7%). Follow-up data were available for 203 patients from the possible FH group and 243 patients from the unlikely FH group. High intensity statin intake status (%) of possible FH vs. unlikely FH was as follows: 1) on admission: 4.8% vs. 0.4%; 2) at discharge: 10.4% vs. 1.6% and 3) at one year follow-up: 5.4% vs. 0.8%. A significantly low percentage of possible FH patients (18.7% vs. 51.4%) achieved target LDL-C levels. There were no significant differences in MACE defined as a composite of cardiogenic shock or Class IV heart failure, recurrent MI, cardiovascular-related rehospitalization, TLR and CV death between the two groups. However, the proportion of cardiogenic shock or Class IV heart failure was significantly higher in possible FH patients group (5.9% vs.1.2%).

**Conclusion:**

Clinical diagnosis of possible FH is common in Chinese patients with premature STEMI. A low proportion of FH patients were prescribed high intensity statins. Despite aggressive cholesterol-lowering drugs, a significantly lower proportion of FH patients achieved LDL-C targets compared to unlikely FH patients. Possible FH patients were younger with a significantly higher occurrence of multi-vessel CAD and impaired cardiac function.

## Introduction

Familial Hypercholesterolemia (FH) is considered to be a genetic disorder of lipid metabolism attributed to defects in the *LDL-receptor* (*LDLR*), *apolipoprotein B* (*APOB*), *proprotein convertase subtilisin/kexin type 9* (*PCSK9*), and *LDL receptor adaptor protein 1* (*LDLRAP1*)[1]. It is characterized by impaired metabolism of low-density lipoprotein-cholesterol (LDL-C) causing severe hypercholesterolemia and consequently leading to accelerated atherosclerosis and premature coronary artery diseases (CAD)[2]. The prevalence of FH in the general population is now estimated to be between 1 in 200 ~ 300 [3–5]. While genetic evaluation can confirm a diagnosis of FH, clinical estimation based on measured cholesterol (mainly LDL-C) levels, physical examination (presence of tendon xanthomas, xanthelasmas and corneal arcus), personal and familial history of CAD is also widely accepted. Recent data show that clinical diagnosis of FH is relatively common in hospitalized patients with premature acute coronary syndrome (ACS) [6–10].

Patients with FH have a 20-fold increased lifetime risk of CAD compared to the general population, if FH is not identified and treated at an early age [11]. Lifestyle modification and appropriate lipid-lowering therapy are recommended and have proved their contribution in reducing this risk[12]. However, FH remains widely underdiagnosed and undertreated worldwide [13, 14] thereby predisposing FH patients to a higher risk in CAD. Clinical trials support the effectiveness and safety of statins in decreasing CAD events in both primary and secondary settings [15] and intensive lipid lowering treatment for FH patients decreases the risk of developing CAD and onset of myocardial infarction (MI) [16]. The adverse effects of FH have also been recognized in China. However, few studies have focused on lipid management and follow up outcomes specifically in FH patients and thus, strategies that can be most effective and efficient in promoting lipid goal attainment for FH patients remain unknown.

This study aimed to investigate the prevalence of clinical FH among Chinese patients with premature ST-segment-elevation myocardial infarction (STEMI) and one-year follow-up on their lipid management and cardiovascular events.

## Materials and methods

### Study population

Our study complied with the Declaration of Helsinki and was approved by the hospital’s ethical review board (Shanghai Tenth People’s Hospital, Tongji University, Shanghai, China). Informed written consent was obtained from all patients enrolled in this study. STEMI patients at <55 years of age for male and <60 years of age for female admitted to Shanghai Tenth People’s Hospital and Chongming Second People’s Hospital were consecutively enrolled from January 2013 to October 2015. The diagnosis of STEMI was based on the presence of characteristic symptoms of myocardial ischemia in association with persistent electrocardiographic ST- segment elevation in at least two contiguous leads and subsequent release of biomarkers of myocardial necrosis.

Patients with significant hematologic disorders, infectious or systemic inflammatory diseases, thyroid dysfunction, severe liver and/or renal insufficiency, and malignant disease were excluded.

All patients underwent cardiac catheterization with stent implantation during hospitalization and significant coronary artery stenosis was defined as >50% reduction in lumen diameter of any of the three coronary arteries or their main branches. Troponin I, creatine kinase-MB (CK-MB) and N-terminal pro-brain natriuretic peptide (NT-proBNP) were measured for every patient on admission. Peripheral blood samples were collected within 24h from patients for assessing fasting lipids, apolipoproteins, lipoprotein (a) [LP (a)], glucose and high sensitive CRP (hs-CRP) levels. Smoking habit, Hypertension, Diabetes mellitus (DM) and body mass index (BMI) were also evaluated. All clinical data were collected via medical records or direct interview of the patients by trained nurses.

### Diagnosis of Familial hypercholesterolemia

Clinical FH was diagnosed using the Dutch Lipid Clinic Network Criteria (DLCN) criteria including personal and family history of premature atherosclerosis, LDL-C levels and xanthomas.

Trained cardiologists examined the cutaneous or tendous xanthomas from the skins and joints of the patients. Patients on lipid-lowering medications with their pretreatment LDL-C unavailable had their untreated LDL-C levels conservatively adjusted by a relative correction factor, here 1.43, which depended on their dose and potency of statins [17].

Based on the DLCN criteria, numerical scores were assigned as follows: (1) family history of a first-degree relative with known premature CAD or vascular disease (<55 years for men, <60 years for women) (1 point) and/or a first-degree relative with known hypercholesterolemia (1 point) or xanthomas (2 points) or offspring(s) with known hypercholesterolemia (2 points). (2) personal history of premature CAD (ages as above, 2 points) or cerebral/peripheral vascular disease (ages as above, 1 point) or xanthomas (6 points); untreated LDL-C>8.5 mmol/L (8 points), 6.5~8.4 mmol/L (5 points), 5.0~6.4 mmol/L (3 points), or 4.0~4.9 mmol/L (1 point); (3) corneal arcus and genetic diagnosis were not available, and these missing information were counted as zero. Finally, a diagnosis of definite FH was considered if the total score was >8 points, probable if the score was 6~8 points, possible if the score was 3~5 points and unlikely if the score was <3 points.

### Follow-up

After discharge all patients were followed-up for 12 months by trained cardiologists at Shanghai Tenth People’s Hospital and Chongming Second People’s Hospital. Those unable to attend their appointment were followed by an interview on the phone. If any patient reported an admission due to recurrent coronary event, they were asked to bring or send by fax the discharge summary. Additionally, all patients were contacted by telephone to assess their clinical status. If patients were not found, information was obtained through family members or patients' treating physician. The primary end point was major adverse cardiac events (MACE) defined as the composite of cardiogenic shock or Class IV heart failure, recurrent MI, cardiovascular-related rehospitalization, target lesion revascularization (TLR) and cardiovascular death.

### Statistical analysis

The values were expressed as mean±SD or median (interquartile range) for continuous variables and number (percentage) for categorical variables. Differences in clinical and biochemical parameters between groups were analyzed using independent t test, Mann–Whitney U test, and Chi-squared tests where appropriate. A P value <0.05 was considered statistically significant. The statistical analysis was performed with SPSS version 22.0 software (SPSS Inc., Chicago, IL).

## Results

Four hundred and ninety-eight premature STEMI patients (363men) were enrolled. During data collection and analysis, of 14 patients who were unable to provide clear family history as they had deceased first degree relatives without available cause of death, 1 patient was from the definite/probable FH group (≥6points), 11 patients were from the possible FH group (3~5 points) and 2 from the unlikely FH group (<3 points). According to the DLCN criteria, the prevalence of definite/probable FH was 3.8% (19 in 498 patients), possible FH was 42.4% (211 in 498 patients) and that of unlikely FH was 53.8% (268 in 498 patients). As only 19 definite/probable FH were identified, its data was merged with possible FH group for further statistical analysis and all patients were divided into two main groups according to the score obtained: unlikely FH (0~2 points) and possible FH (≥3 points). Unavailability of family history of those 12 patients (1 from definite/probable FH group and 11 from the possible FH group) does not affect their group classification as they already have a score of ≥3 points regardless of family history.

The baseline characteristics of the patients according to FH diagnosis are shown in Table 1.

**Table 1:** Baseline characteristics of the study population.

	Possible FH (n = 230)	Unlikely FH (n = 268)	P value
Age (y)	50.1±3.9	53.5±4.2	<0.001
Men, % (n)	72.6(167)	73.1(196)	0.981
Current smokers, % (n)	60.4(139)	62.7(168)	0.664
Former smokers, % (n)	9.6(22)	8.2(22)	0.696
Hypertension, % (n)	50.0(115)	51.5(138)	0.807
Diabetes mellitus, % (n)	25.7(59)	23.9(64)	0.719
Family history of CAD, % (n)	62.2(143)	0.7(2)	<0.001
Systolic Blood Pressure(mmHg)	132±12.9	130±14.1	0.101
Diastolic Blood Pressure(mmHg)	74±10.0	75±8.6	0.231
Body mass index (kg/m^2^)	25.6±3.7	25.2±3.4	0.210
Hypertriglyceridemia, % (n)	34.8(80)	25.4(68)	0.029
Laboratory analysis			
Total cholesterol (mmol/L)	7.38±0.93	4.10±1.02	<0.001
Triglycerides (mmol/L)	1.72±1.31	1.67±1.14	0.649
HDL cholesterol (mmol/L)	0.98±0.28	1.03±0.38	0.010
LDL cholesterol (mmol/L)	5.77±0.36	2.69±0.41	<0.001
Apolipoprotein A-I (mg/dL)	123.1±21.3	119.6±22.9	0.080
Apolipoprotein B (mg/dL)	112.3±25.8	88.7±25.3	<0.001
Lipoprotein(a) (mg/dL)	37.0±18.8	33.4±17.1	0.026
Glucose (mmol/L)	5.81±1.21	6.0±1.30	0.094
HbA1C (%)	6.33±1.12	6.24±1.41	0.436
Troponin I(μg/L)	201.33±21.54	199.35±19.87	0.287
CK-MB (μg/L)	224.51±22.97	220.33±26.85	0.065
NT-proBNP(pg/mL)	3014.15±281.74	2326.25±212.94	<0.001
Hs-CRP (mg/L)	1.71(0.85–7.14)	1.58(0.41–5.37)	0.138
Multivessel CAD, % (n)	37.4(86)	18.3(49)	<0.001
Left ventricle ejection fraction (%)	47±9	49 ± 6	0.003
Killip class, % (n)			
1	53.0(122)	69.8(187)	<0.001
2	23.9(55)	19.8(53)	0.318
3	20.0(46)	9.7(26)	0.002
4	3.0(7)	0.7(2)	0.109
CV death, % (n)	3.5(8)	3.7(10)	0.903

Abbreviations: CAD, coronary artery disease; HDL, high density lipoprotein; LDL, low density lipoprotein; HbA1C, hemoglobin A1c; CK-MB, ceatine kinase-MB; NT-proBNP, N-terminal pro-brain natriuretic peptide; Hs-CRP, high sensitive CRP; CV, cardiovascular.

There was no significant difference between the groups when factors such as sex, smoking habits, hypertension or DM were considered. Possible FH patients were younger (50.1 years vs. 53.5 years) and had higher proportion of family history of premature CAD (62.2% vs. 0.7%). Patients with possible FH had higher total cholesterol, LDL-C, triglycerides and Lp(a) levels and were more likely to develop multivessel CAD (37.4% vs. 18.3%) compared to the unlikely FH patients. There was no significant difference between the groups in levels of Troponin I and CK-MB while possible FH patients had significantly higher NT-proBNP level (3014.15 pg/mL vs. 2326.25 pg/mL), lower LVEF (47% vs. 49%) and more severe Killip classification (Class 3, 20.0% vs. 9.7%). During the hospitalization period no recurrent MI and TLR occurred in all patients. There was no significant difference in the CV deaths between the groups.

The cholesterol-lowering medication intake status of the patients is described in Table 2. The high intensity statin intake status % (n) [Ezetimibe % (n)] of the possible FH group vs. unlikely FH group was as follows: 1) on admission 4.8% (11) [0.9% (2)] vs. 0.4% (1) [0.4% (1)]; 2) at discharge, 10.4% (23) [2.3% (5)] vs. 1.6% (4) [0.8% (2)] and 3) one-year follow-up 5.4% (11) [2.0% (4)] vs. 0.8% (2) [0.8% (2)].

**Table 2:** Cholesterol-lowering medication data of patients at different intervals.

Cholesterol-lowering medication, % (n)	on admission		at discharge		1 year follow-up	
	Possible FH(n = 230)	Unlikely FH(n = 268)	Possible FH(n = 222)	Unlikely FH(n = 258)	Possible FH(n = 203)	Unlikely FH(n = 243)
Low intensity	1.7(4)	7.5(20)	0(0)	0.4(1)	1.5(3)	5.3(13)
Moderate intensity	77.8(179)	69.8(187)	86.5(192)	96.9(250)	90.6(184)	92.6(225)
High intensity*	4.8(11)	0.4(1)	10.4(23)	1.6(4)	5.4(11)	0.8(2)
Off treatment	14.8(34)	20.9(56)	0.9(2)	0.4(1)	0.5(1)	0.4(1)
Ezetimibe	0.9(2)	0.4(1)	2.3(5)	0.8(2)	2.0(4)	0.8(2)

*Atorvastatin 40–80 mg or rosuvastatin 20–40 mg

One year after their cardiovascular event, 203 patients from the possible FH group and 243 patients from the unlikely FH group came for follow-up. LDL-C and TC levels of both groups decreased significantly. Despite being treated more aggressively with cholesterol-lowering drugs, a lower percentage of possible FH patients (18.7%) achieved targeted LDL-C levels (LDL-C <1.8mmol/L or a decrease >50% of the LDL-C levels on admission) compared to 51.4% of unlikely FH patients. At 1 year follow-up, the two groups showed no significant difference in MACE which was defined as a composite of cardiogenic shock or Class IV heart failure, recurrent MI, cardiovascular-related rehospitalization, TLR and CV death. However, the proportion of cardiogenic shock or Class IV heart failure was significantly higher in possible FH patients group (5.9% vs.1.2%). Recurrent MI occurred in 3 of the possible FH group and all 3 were from the 19 definite/probable FH patients while only 1 case occurred in the unlikely FH group. The proportion of TLR in the possible FH group was 3.4% compared to 1.2% in the unlikely FH group and although there was no significant difference between the groups, possible FH group showed an increasing trend in TLR. Cardiovascular-related rehospitalization and CV death had no significant differences between the two groups. The data is shown in Table 3.

**Table 3:** One-year follow-up data of patients.

	Possible FH (n = 203)	Unlikely FH (n = 243)	P value
Body mass index (kg/m^2^)	24.0±2.1	23.7±2.4	0.165
Laboratory analysis			
Total cholesterol (mmol/L)	5.54±1.21	3.89±0.98	<0.001
Triglycerides (mmol/L)	1.65±0.92	1.54±1.02	0.236
HDL cholesterol (mmol/L)	1.10±0.46	1.18±0.59	0.116
LDL cholesterol (mmol/L)	3.47±0.97	2.11±0.88	<0.001
Apolipoprotein A-I (mg/dL)	117.4±20.1	114.8±17.3	0.142
Apolipoprotein B (mg/dL)	90.0±18.2	86.0±15.4	0.012
Lipoprotein(a) (mg/dL)	30.1±2.9	29.7±3.1	0.163
<1.8mmol/L or a decrease >50% of initial LDL-C levels available on admission, % (n)	18.7 (38)	51.4 (125)	<0.001
Cardiogenic shock or Class IV heart failure, % (n)	5.9 (12)	1.2 (3)	0.013
Recurrent MI, % (n)	1.5 (3)	0.4 (1)	0.471
Cardiovascular-related rehospitalization, % (n)	6.4 (13)	4.5 (11)	0.499
TLR, % (n)	3.4 (7)	1.2 (3)	0.210
CV death, % (n)	4.9 (10)	4.5 (11)	0.979
MACE, % (n)	9.4 (19)	6.2 (15)	0.277

Abbreviations: HDL, high density lipoprotein; LDL, low density lipoprotein; MI, myocardial infarction; TLR, target lesion revascularization; CV, cardiovascular; MACE, major adverse cardiovascular events.

## Discussion

Our study investigated the status of clinical diagnosis of FH in Chinese patients with premature STEMI and one-year follow-up on their lipid management and cardiovascular events. The major findings were 1) Clinical diagnosis of possible FH is relatively common in Chinese patients with premature STEMI, 2) A low proportion of FH patients were prescribed high intensity statins. Despite aggressive cholesterol-lowering drugs, a significantly lower proportion of FH patients achieved LDL-C targets compared to unlikely FH patients, 3) Possible FH patients were younger with significantly higher occurrence of multi-vessel CAD and impaired cardiac function.

Phenotypic diagnosis of FH is more appropriate for clinical use as genetic screening is not easily endorsed in the clinical setup. It is only after the occurrence of their first cardiovascular event that FH patients can be identified based on lipid profiles and diagnostic criteria such as the DLCN algorithm and the Simon Broome criteria. Therefore, the hospital is considered as a useful setup in the identification of FH. STEMI in young patients is a life threatening condition and identification of FH in this subpopulation is of vital importance as premature ACS is one of the important clinical manifestations of the disease. Recently, studies have investigated the prevalence of FH in patients with premature ACS. The reported prevalence of phenotypic diagnosis of FH based on the DLCN algorithm varies from study to study due to the cut-off age used to define AMI. A study by Rallidis et al. which investigated the prevalence of FH in patients <35 years with first STEMI identified 20.3% with definite/probable FH and 50.9% with possible FH[7]. In a larger multicenter cohort study in Switzerland among 1451 young patients with premature ACS, 4.8% had probable/definite FH and 47.1% had possible FH [6]. From a larger population, Mortensen et al. identified 6.9% probable/definite FH patients among the 291 (21%) patients with premature MI[18]. The EUROASPIRE IV study [19] also found a high prevalence of FH among patients with premature coronary heart diseases (<50 years) while a smaller study by Pang et al. [8] showed that the prevalence of probable/definite FH was 14.3% among premature CAD patients in a CCU setting. A similar trend was seen in a Chinese study in which 48.2% of the study population had premature MI and when the prevalence of FH was assessed, 7.1% of them were diagnosed with definite/probable FH [10]. These data reflect the increasing burden of FH and therefore emphasizes the need for mandatory phenotypic screening of FH in such populations. Results from our study, together with other studies carried out on the Chinese population show that FH is not rare in the Chinese population [17] but is undertreated and underdiagnosed. The prevalence of definite/probable FH in our present study was found to be 3.8% and is lower than the percentage reported by Li et al [10]. This disparity could be explained by the poor availability of information about the family history of some of our patients. Of 14 patients who were unable to provide clear family history as they had deceased first degree relatives without available cause of death, 11 patients were from the possible FH group (3~5 points). Availability of family history about these possible FH patients could lead to some of them to be in the definite/probable group thereby increasing the prevalence of definite/probable FH.

The 2013 European Atherosclerosis Society recommends high intensity statins in treating FH and if necessary supplemented by Ezetimibe [13]. Our results showed that a relatively low proportion of FH patients were given high intensity statins and although they were treated more aggressively with cholesterol-lowering drugs, the control of LDL-C levels in FH patients is poor compared with patients with unlikely FH. Several reasons might account for the low percentage of FH patients achieving recommended LDL-C levels in the present study. The first was under diagnosis of FH by physicians due to their lack of knowledge about the disease and the treatment of underdiagnosed FH patients as usual STEMI patients in such cases. The second reason might be that FH patients were not treated with intensive LDL-C lowering medications due to lack of knowledge of physicians about intensive LDL-C lowering drugs and concern of side effects of drugs. These can explain for the low percentage of patients being on high intensity statin and Ezetimibe on admission and a slightly higher percentage at discharge. A third but equally important reason might be non-compliance in Chinese patients, who were not taking the required doses of cholesterol-lowering drugs as prescribed and did not implement any lifestyle modifications such as a diet low in cholesterol and exercise. This can be seen in the one-year follow-up by the decreased rates of possible FH patients being on high intensity therapy and Ezetimibe as compared to the prescribed doses at discharge. In addition, it has been seen that despite being on maximum doses of statin with the addition of Ezetimibe, a low percentage ranging from 10.4~22% of heterozygous FH patients reached the less stringent levels of <2.5mmol/L of LDL-C levels [20, 21]. A previous study showed that there was no difference in intensive vs. standard statin treatment in patients with elevated LDL-C levels in Asian population[22] and increasing the statin dose by two-fold had only shown a further 6.4% decrease in LDL-C levels in Chinese population[23]. Accordingly, a low proportion of FH patients achieved LDL-C targets despite statin treatments.

FH causes severe hypercholesterolemia consequently leading to accelerated atherosclerosis and premature CAD as individuals with FH are born with a high LDL-C cholesterol level in contrast to those who acquire hypercholesterolemia later in life. Clinical FH among patients with first MI was strongly related with MI occurring prematurely by as much as 15 years[18] and 10 years[10]. We also found a significant difference between the ages of onset of premature MI between the two groups with MI occurring 2~3 years earlier in possible FH group. Our study also showed that patients with possible FH exhibited a higher prevalence of multivessel CAD confirming the critical role of FH in the development of atherosclerosis [24]. However, the present data did not show any significant difference in MACE, recurrent MI, cardiovascular-related rehospitalization, TLR and CV death between the two groups at one-year follow-up. A previous study showed no significant differences in the rate of recurrent MI of the first two years and all-cause mortality in a median follow-up of 3.3 years in premature STEMI patients diagnosed with FH[9]. However, in the same study, the unadjusted and adjusted event rates of recurrent MI were higher in patients with possible FH compared with unlikely FH after the first two years[9]. In the present study, all 3 recurrent MI in possible FH group were from 19 definite/probable FH patients. The proportion of TLR was 3.4% in possible FH group compared to 1.2% in unlikely FH group showing an increasing trend in TLR in possible FH patients. In addition, possible FH patients had higher levels of NT-proBNP on admission and impaired cardiac function both on admission and at one year follow-up. Higher levels of NT-proBNP[25] and impaired cardiac function both represent a higher risk of subsequent adverse cardiac events. Based on these data, we educe that there would be a significant difference in the incidence of MACE between the two groups with the extension of follow-up.

### Limitations

Several limitations need to be considered in the present study. First, we did not use the criteria relating to corneal arcus and molecular genetic testing for FH identification. Second, the LDL-C levels for FH diagnosis might have a certain bias: we used the estimated values rather than the true untreated LDL-C for the medical-treated patients; MI status has been demonstrated to generate changes in levels of circulating cholesterol known as the acute phase response. In addition, small sample size, short period of follow-up and loss of follow-up could contribute to a bias of the present result in our study.

## Conclusion

Clinically diagnosed FH is relatively common in Chinese patients with premature STEMI. A low proportion of FH patients were prescribed high intensity statins. Despite aggressive cholesterol-lowering drugs, a significantly lower proportion of FH patients achieved LDL-C targets compared to unlikely FH patients. Possible FH patients were younger with a significantly higher occurrence of multi-vessel CAD and impaired cardiac function. There was no difference in MACE between two groups at one-year follow-up, thus emphasizing the need for continuous follow-up.

## Supporting information

S1 Checklist STROBE-checklist-cohort. (DOC)
