# Supplementary material for: Familial hypercholesterolemia in Chinese patients with premature ST-segment-elevation myocardial infarction: Prevalence, lipid management and 1-year follow-up
Source: PLoS One. 2017 Oct 31;12(10):e0186815. doi: 10.1371/journal.pone.0186815 (PMC5663389; doi:10.1371/journal.pone.0186815)
Supplement: S1 Checklist — (DOC) [file pone.0186815.s001.doc]

STROBE Statement—Checklist of items that should be included in reports of ***cohort studies***

|  | Item No. | Recommendation | Page No. | Relevant text from manuscript |
| --- | --- | --- | --- | --- |
| **Title and abstract** | 1 | (*a*) Indicate the study’s design with a commonly used term in the title or the abstract | 1 | Prevalence, Lipid Management and 1-year follow-up |
|  |  | (*b*) Provide in the abstract an informative and balanced summary of what was done and what was found | 2-3 | We investigated the prevalence of clinical FH among premature STEMI patients and one-year follow-up on lipid management and cardiovascular events.  Possible FH is common in Chinese premature STEMI patients. A low proportion of FH patients were prescribed high intensity statins and achieved LDL-C targets. Possible FH patients were younger with a significantly higher occurrence of multi-vessel CAD and impaired cardiac function. |
| Introduction | | |  |  |
| Background/rationale | 2 | Explain the scientific background and rationale for the investigation being reported | 4 | Few studies have focused on lipid management and follow up outcomes specifically in FH patients. |
| Objectives | 3 | State specific objectives, including any prespecified hypotheses | 4 | This study aimed to investigate the prevalence of clinical FH among Chinese premature STEMI patients and one-year follow-up on their lipid management and cardiovascular events. |
| Methods | | |  |  |
| Study design | 4 | Present key elements of study design early in the paper | 4 | Premature STEMI patients were consecutively enrolled and were followed-up for 12 months. |
| Setting | 5 | Describe the setting, locations, and relevant dates, including periods of recruitment, exposure, follow-up, and data collection | 4,5,6 | Patients admitted to Shanghai Tenth People’s Hospital and Chongming Second People’s Hospital were consecutively enrolled from January 2013 to October 2015. All clinical data were collected via medical records or direct interview of the patients by trained nurses. |
| Participants | 6 | (*a*) Give the eligibility criteria, and the sources and methods of selection of participants. Describe methods of follow-up | 4-6 | STEMI patients at <55 years of age for male and <60 years of age for female admitted to Shanghai Tenth People’s Hospital and Chongming Second People’s Hospital were consecutively enrolled from January 2013 to October 2015. Clinical FH was diagnosed using the Dutch Lipid Clinic Network Criteria (DLCN) criteria. After discharge all patients were followed-up for 12 months by trained cardiologists. |
| Variables | 7 | Clearly define all outcomes, exposures, predictors, potential confounders, and effect modifiers. Give diagnostic criteria, if applicable | 5 | Troponin I, CK-MB, NT-proBNP, lipids, apolipoproteins, LP, glucose and hs-CRP were assessed. Smoking habit, Hypertension, DM and BMI were also evaluated. Clinical FH was diagnosed using the DLCN criteria. |
| Data sources/ measurement | 8* | For each variable of interest, give sources of data and details of methods of assessment (measurement). Describe comparability of assessment methods if there is more than one group | *5,8* | Troponin I, CK-MB, NT-proBNP, lipids, apolipoproteins, LP, glucose and hs-CRP were assessed. Smoking habit, Hypertension, DM and BMI were also evaluated.  Table 1. |
| Bias | 9 | Describe any efforts to address potential sources of bias | 5 | Patients with hematologic disorders, infectious or systemic inflammatory diseases, thyroid dysfunction, severe liver and/or renal insufficiency, and malignant disease were excluded. |
| Study size | 10 | Explain how the study size was arrived at | 4 | STEMI patients at <55 years of age for male and <60 years of age for female admitted to Shanghai Tenth People’s Hospital and Chongming Second People’s Hospital were consecutively enrolled from January 2013 to October 2015. |
| Quantitative variables | 11 | Explain how quantitative variables were handled in the analyses. If applicable, describe which groupings were chosen and why | 6 | Differences in clinical and biochemical parameters between groups were analyzed using independent t test, Mann–Whitney U test, and Chi-squared tests where appropriate. |
| Statistical methods | 12 | (*a*) Describe all statistical methods, including those used to control for confounding | 6 | Differences in clinical and biochemical parameters between groups were analyzed using independent t test, Mann–Whitney U test, and Chi-squared tests where appropriate |
|  |  | (*b*) Describe any methods used to examine subgroups and interactions |  |  |
|  |  | (*c*) Explain how missing data were addressed | 9,12 | One year after their cardiovascular event, 203 patients from the possible FH group and 243 patients from the unlikely FH group came for follow-up. Of 14 patients who were unable to provide clear family history as they had deceased first degree relatives without available cause of death, 11 patients were from the possible FH group. |
|  |  | (*d*) If applicable, explain how loss to follow-up was addressed |  |  |
|  |  | (*e*) Describe any sensitivity analyses |  |  |
| Results | | |  |  |
| Participants | 13* | (a) Report numbers of individuals at each stage of study—eg numbers potentially eligible, examined for eligibility, confirmed eligible, included in the study, completing follow-up, and analysed | 7,9 | Four hundred and ninety-eight premature STEMI patients (363men) were enrolled. One year after their cardiovascular event, 203 patients from the possible FH group and 243 patients from the unlikely FH group came for follow-up. |
|  |  | (b) Give reasons for non-participation at each stage |  |  |
|  |  | (c) Consider use of a flow diagram |  |  |
| Descriptive data | 14* | (a) Give characteristics of study participants (eg demographic, clinical, social) and information on exposures and potential confounders | 8 | Table 1. |
|  |  | (b) Indicate number of participants with missing data for each variable of interest | 7 | During data collection and analysis, of 14 patients who were unable to provide clear family history as they had deceased first degree relatives without available cause of death, 1 patient was from the definite/probable FH group (≥6points), 11 patients were from the possible FH group (3~5 points) and 2 from the unlikely FH group (<3 points).. |
|  |  | (c) Summarise follow-up time (eg, average and total amount) | 9 | One year after their cardiovascular event, 203 patients from the possible FH group and 243 patients from the unlikely FH group came for follow-up. |
| Outcome data | 15* | Report numbers of outcome events or summary measures over time | 8,9,10 | Table 1,2,3 |
| Main results | 16 | (*a*) Give unadjusted estimates and, if applicable, confounder-adjusted estimates and their precision (eg, 95% confidence interval). Make clear which confounders were adjusted for and why they were included |  |  |
|  |  | (*b*) Report category boundaries when continuous variables were categorized |  |  |
|  |  | (*c*) If relevant, consider translating estimates of relative risk into absolute risk for a meaningful time period |  |  |
| Other analyses | 17 | Report other analyses done—eg analyses of subgroups and interactions, and sensitivity analyses |  |  |
| Discussion | | |  |  |
| Key results | 18 | Summarise key results with reference to study objectives | 11 | The major findings were 1) Clinical diagnosis of possible FH is relatively common in Chinese patients with premature STEMI, 2) A low proportion of FH patients were prescribed high intensity statins. Despite aggressive cholesterol-lowering drugs, a significantly lower proportion of FH patients achieved LDL-C targets compared to unlikely FH patients, 3) Possible FH patients were younger with significantly higher occurrence of multi-vessel CAD and impaired cardiac function. |
| Limitations | 19 | Discuss limitations of the study, taking into account sources of potential bias or imprecision. Discuss both direction and magnitude of any potential bias | 14,15 | Several limitations need to be considered in the present study. First, we did not use the criteria relating to corneal arcus and molecular genetic testing for FH identification. Second, the LDL-C levels for FH diagnosis might have a certain bias: we used the estimated values rather than the true untreated LDL-C for the medical-treated patients; MI 268 status has been demonstrated to generate changes in levels of circulating cholesterol known as the acute phase response. In addition, small sample size, short period of follow-up and loss of follow-up could contribute to a bias of the present result in our study. |
| Interpretation | 20 | Give a cautious overall interpretation of results considering objectives, limitations, multiplicity of analyses, results from similar studies, and other relevant evidence | 15 | Clinically diagnosed FH is relatively common in Chinese patients with premature STEMI. A low proportion of FH patients were prescribed high intensity statins. Despite aggressive cholesterol lowering drugs, a significantly lower proportion of FH patients achieved LDL-C targets compared to unlikely FH patients. Possible FH patients were younger with a significantly higher occurrence of multi-vessel CAD and impaired cardiac function. There was no difference in MACE between two groups at one-year follow-up, thus emphasizing the need for continuous follow-up. |
| Generalisability | 21 | Discuss the generalisability (external validity) of the study results | 14,15 | Based on these data, we educe that there would be a significant difference in the incidence of MACE between the two groups with the extension of follow-up.  There was no difference in MACE between two groups at one-year follow-up, thus emphasizing the need for continuous follow-up. |
| Other information | | |  |  |
| Funding | 22 | Give the source of funding and the role of the funders for the present study and, if applicable, for the original study on which the present article is based |  | This study was supported by National Natural Science Foundation of China (81570436), Foundation of Shanghai Municipal Commission of Health and Family Planning (201640053), Shanghai Medical Guiding Project (124119a6900). |

*Give information separately for exposed and unexposed groups.

**Note:** An Explanation and Elaboration article discusses each checklist item and gives methodological background and published examples of transparent reporting. The STROBE checklist is best used in conjunction with this article (freely available on the Web sites of PLoS Medicine at http://www.plosmedicine.org/, Annals of Internal Medicine at http://www.annals.org/, and Epidemiology at http://www.epidem.com/). Information on the STROBE Initiative is available at http://www.strobe-statement.org.
